# Genomic Editing of Non-Coding RNA Genes with CRISPR/Cas9 Ushers in a Potential Novel Approach to Study and Treat Schizophrenia

**DOI:** 10.3389/fnmol.2017.00028

**Published:** 2017-02-03

**Authors:** Chuanjun Zhuo, Weihong Hou, Lirong Hu, Chongguang Lin, Ce Chen, Xiaodong Lin

**Affiliations:** ^1^Department of Psychiatry, Wenzhou Seventh People’s HospitalWenzhou, China; ^2^Department of Psychiatry, Tianjin Mental Health Center, Tianjin Anding HospitalTianjin, China; ^3^Department of Psychiatry, Tianjin Anning HospitalTianjin, China; ^4^Department of Biology, University of North Carolina at CharlotteCharlotte, NC, USA

**Keywords:** schizophrenia, non-coding RNAs, lncRNAs, miRNAs, CRISPR/Cas9, gene editing

## Abstract

Schizophrenia is a genetically related mental illness, in which the majority of genetic alterations occur in the non-coding regions of the human genome. In the past decade, a growing number of regulatory non-coding RNAs (ncRNAs) including microRNAs (miRNAs) and long non-coding RNAs (lncRNAs) have been identified to be strongly associated with schizophrenia. However, the studies of these ncRNAs in the pathophysiology of schizophrenia and the reverting of their genetic defects in restoration of the normal phenotype have been hampered by insufficient technology to manipulate these ncRNA genes effectively as well as a lack of appropriate animal models. Most recently, a revolutionary gene editing technology known as Clustered Regularly Interspaced Short Palindromic Repeats (CRISPR)/CRISPR-associated nuclease 9 (Cas9; CRISPR/Cas9) has been developed that enable researchers to overcome these challenges. In this review article, we mainly focus on the schizophrenia-related ncRNAs and the use of CRISPR/Cas9-mediated editing on the non-coding regions of the genomic DNA in proving causal relationship between the genetic defects and the pathophysiology of schizophrenia. We subsequently discuss the potential of translating this advanced technology into a clinical therapy for schizophrenia, although the CRISPR/Cas9 technology is currently still in its infancy and immature to put into use in the treatment of diseases. Furthermore, we suggest strategies to accelerate the pace from the bench to the bedside. This review describes the application of the powerful and feasible CRISPR/Cas9 technology to manipulate schizophrenia-associated ncRNA genes. This technology could help researchers tackle this complex health problem and perhaps other genetically related mental disorders due to the overlapping genetic alterations of schizophrenia with other mental illnesses.

## Introduction

Schizophrenia is a severe mental illness, in which patients exhibit both mental and behavioral dysfunction mainly associated with neurodevelopmental abnormality. Indeed, schizophrenia is considered as a neurodevelopmental disorder (Marenco and Weinberger, 2000). To date, the pathological causes underlying the onset and progression of schizophrenia remain unclear.

A wealth of science-based evidence has indicated that schizophrenia is a genetically related mental illness that is inherited in approximately 80% of cases (Cardno et al., 1999) and that certain alterations in the genomic DNA inherited from the parents are likely to contribute to disease onset in late adolescence or early adulthood (International Schizophrenia Consortium et al., 2009; Costain et al., 2013; *Human Molecular Genetics*). Decades of research have shown that the interplay between genes and environmental factors largely contribute to the development of schizophrenia (Owen et al., 2016). It has been found that the majority of genetic alterations occur in the non-coding regions of human genome, from which regulatory non-coding RNAs (ncRNAs), mainly microRNAs (miRNAs) and long non-coding RNAs (lncRNAs), are transcribed. miRNAs and lncRNAs are two major regulatory ncRNAs with little capacity to encode proteins and have differences in size, location and biological function. miRNAs play a vital role in the modulation of gene expression, mainly via post-transcriptional destabilization of target mRNAs that harbor one or more complementary sites with miRNAs, translational repression, or a combination of the two molecular mechanisms (Bartel, 2004, 2009). While lncRNAs are defined as regulatory ncRNAs with a size longer than 200 nt in length. In contrast to miRNAs, our knowledge with respect to the molecular mechanism whereby lncRNAs function remains largely unexplored (Ponting et al., 2009; Wilusz et al., 2009). Recently, a growing number of lncRNAs have been reported to be associated with a broad range of diseases, including mental illnesses such as schizophrenia (Barry et al., 2014; Merelo et al., 2015; Rao et al., 2015). miRNAs and lncRNAs as two large groups of regulatory ncRNAs are highly expressed in the brain where they act as important modulators of genes essential for maintaining proper brain development and function. This is of particular importance because it has been well documented that abnormal brain development, maturation and plasticity have been strongly linked to schizophrenia. For instance, manipulation of schizophrenia-associated miRNAs such as miR-132/miR-121 and miR-219 has been reported to alter an activity-dependent neuronal maturation and plasticity at synapse (Nudelman et al., 2010; Tognini et al., 2011; Mellios and Sur, 2012). Further studies have shown that these miRNAs function through either directly regulating synthesis of proteins which are essential for plasticity at the synapse or interacting with transcription factors which are likely to modulate more enduring neuroplastic changes (Smalheiser and Lugli, 2009; Mellios and Sur, 2012). These recent findings of schizophrenia-associated miRNAs suggest that the biological implication of dysregulation of the miRNAs and their targeted genes are profound for schizophrenia. Considering the magnitude of miRNA alterations and their broad effects on target genes in schizophrenia, the schizophrenia-associated miRNAs might be important for the pathogenesis of schizophrenia. Over past years, an increasing number of schizophrenia-associated ncRNAs such as miR-137, lncRNA Gomafu, etc., has been identified, and the correlation of their genetic alterations with the pathogenesis of schizophrenia has been characterized (Sone et al., 2007; International Schizophrenia Consortium et al., 2009; Schizophrenia Psychiatric Genome-Wide Association Study (GWAS) Consortium, 2011; Barry et al., 2014; Ma et al., 2014). However, the exact functional roles of these genetic variations in the development and progression of schizophrenia are less understood. Indeed, the functioning of these ncRNAs in the development of schizophrenia and the correction of their genetic changes in the genomic DNA, the key to develop novel therapies for schizophrenia, have been hampered by insufficient technology to manipulate these ncRNA genes effectively and a lack of appropriate animal models. Apparently, understanding these changes in the genomic DNA, including the coding and non-coding regions in schizophrenia, in combination with the development of novel genetic tools, will undoubtedly enable the mutations to be corrected, which is the key to developing novel therapies for schizophrenia.

In recent years, with a range of advanced technologies, including next-generation sequencing, high-resolution microarrays and genotyping, a great number of miRNAs and lncRNAs associated with schizophrenia have been identified and characterized. The understanding of these ncRNAs and their genetic variations in the development of schizophrenia has relied on the novel tools of advanced technologies in biology and beyond. Clustered regularly interspaced short palindromic repeats (CRISPR)/CRISPR-associated nuclease 9 (Cas9) is a recently developed revolutionary gene editing technology that is able to manipulate effectively the non-coding regions of the genomic DNA in human cell lines and animal models, to create or correct the mutations more easily than ever in living cells and animals, and therefore to overcome obstacles in schizophrenia research and therapy (Bhaya et al., 2011; Jinek et al., 2012; Cho et al., 2013; Cong et al., 2013; Jiang et al., 2013; Mali et al., 2013; Doudna and Charpentier, 2014).

In this review article, we bring together schizophrenia-associated regulatory ncRNAs, mainly miRNAs and lncRNAs, summarize CRISPR/Cas9-mediated editing on the non-coding regions of genomic DNA in human cell lines and animal models, and propose applications of the revolutionary gene editing CRISPR/Cas9 tool in schizophrenia research, especially on the functional study of ncRNAs genes and on the development of animal models to accelerate schizophrenia research, thus proving the causal relationship between genetic defects and the pathophysiology of schizophrenia, and eventually translating the scientific findings into a clinical cure for schizophrenia. The information described in the present review may help researchers to tackle this complex health problem and perhaps other genetically related mental disorders due to the overlapping genetic alterations of schizophrenia with other mental illnesses.

## Methods

A systematic search and review of the recent literature was performed using the main databases, including PubMed, Google Scholar and Wanfang Med Online, by using the following keywords: schizophrenia, mental disorder, gene editing, CRISPR/Cas9, ncRNAs, miRNAs and lncRNAs.

## ncRNAs Including miRNAs and lncRNAs That Are Associated with Schizophrenia

The involvement of complex genetic components in the etiology of schizophrenia has been well established by a wealth of scientific evidence. Indeed, the heritability of schizophrenia is approximately 80% (Cardno et al., 1999); therefore, novel genomic tools offer hope for insight into the profound etiology of this complicated, genetically related mental illness. Genome-wide association studies have revealed a great number of strong susceptibility loci for schizophrenia, most of which are located in the non-coding regions of the genome, including genetic fragments for the transcription of miRNAs and lncRNAs. In fact, numerous studies have found that the majority of miRNAs and lncRNAs are highly expressed in the brain, where their genetic mutations, abnormal expression and impact on target gene levels may lead to defective development, suppression of synaptic efficacy, and mGluR-dependent synaptic plasticity in the hippocampus (Olde Loohuis et al., 2015; Siegert et al., 2015) as well as other neurological impairment that is largely correlated with schizophrenia and may contribute to its pathogenesis and progression (Krichevsky et al., 2003; Sempere et al., 2004; Giraldez et al., 2005; Vo et al., 2005; Schratt et al., 2006). In the past decade or two, with the help of high-throughput microarray techniques, comparison of differential expression of miRNAs in schizophrenia vs. controls has identified numerous miRNAs that are dysregulated in schizophrenia, which have been summarized previously (Beveridge and Cairns, 2012; Wang J. et al., 2014). In contrast to the above studies with microarray analysis, a genome-wide association study performed in a large-scale population revealed schizophrenia-associated non-coding genes, including miRNA genes, among which miRNA137 stands out (International Schizophrenia Consortium et al., 2009; Schizophrenia Psychiatric Genome-Wide Association Study (GWAS) Consortium, 2011). International Schizophrenia Consortium et al. (2009) reported that the genetic elements for a small ncRNA, the microRNA137 (*MIR137*) gene with chromosomal location 1p21.3, have been the schizophrenia-associated loci, conferring susceptibility to schizophrenia and the discovery has received great attention within the field. In the following large-scale genome-wide association study conducted by Franke et al. (2016) using more than 40,000 recruited participants, the rs1625579 polymorphism in the miRNA137 gene was identified to be strongly linked to schizophrenia (Schizophrenia Psychiatric Genome-Wide Association Study (GWAS) Consortium, 2011; Ma et al., 2014). To date, many further investigations have supported the association between miR137 and schizophrenia as well as the miR-137 variant rs1625579 as a predictor of an earlier age-at-onset of psychosis in schizophrenia (Lett et al., 2013). In a Han Chinese population, analysis of the single nucleotide polymorphism rs1625579 of the miRNA137 gene in a cohort of 1430 individuals with schizophrenia compared with 1570 healthy individuals was performed; rs1625579 showed significant differences in the allele frequencies between those who suffered from schizophrenia and healthy control subjects (Guan et al., 2014), while the single nucleotide polymorphism rs66642155 allelic variant was likely to have an impact on the age-at-onset and the degree of positive symptoms (Wang S. et al., 2014). The molecular mechanism whereby the genetic variants of miR-137 confer a high risk of schizophrenia also has been shown to be related to a declined fronto-striatal brain white and gray matter structure but without changes in the volume of the local brain (Rose et al., 2014; Wright et al., 2016), which may cause symptoms such as poor concentration, low processing speed, cognitive impairment, etc. (Kuswanto et al., 2015); the impact of miR137 genetic alterations on the expression of a set of target neuronal transmission genes and synaptogenesis (Kos et al., 2015; Strazisar et al., 2015; Wright et al., 2015); and involvement of miR137 on a glucocorticoid receptor-dependent signaling pathway (Vallès et al., 2014). A very recent in-depth study in a zebrafish model system by Giacomotto et al. (2016) has reported that a slight change in synaptic function or a defect in the axonal network mediated by schizophrenia-associated suppression of miR-137 is likely responsible for the behavioral phenotype.

Apart from miRNAs, lnRNAs have emerged as one of most important classes of ncRNAs in the regulation of gene expression, and their genetic alterations and dysregulation play an important role in the pathogenesis of various diseases. In contrast to a larger number of schizophrenia-associated miRNAs being studied, only several lncRNAs have been identified to be correlated with this disease, among which myocardial infraction-associated transcript (MIAT), also known as retinal ncRNAs 2, was most recently found to be strongly correlated with schizophrenia. MIAT was originally identified as a new member of the lncRNA family in Blackshaw et al. (2004), and it was initially found to play a role in regulating retinal cell fate differentiation and to be associated with myocardial infraction (Ishii et al., 2006; Rapicavoli et al., 2010; Liao et al., 2016). It is also abundantly expressed in the nucleus of neurons during development through adulthood. In addition, MIAT is known as lncRNA Gomafu (Sone et al., 2007), a Japanese word reflecting its speckled distribution in the nucleoplasm. Despite its chromosomal location of 22q12.1, which has been documented to be associated with schizophrenia, and its expression in the nervous system, the link between this lncRNA and schizophrenia was unveiled in 2014. The Australian research team of Barry et al. (2014) has demonstrated for the first time the strong association of MIAT with schizophrenia, including downregulation of Gomafu in schizophrenic patients, and has provided insight into the mechanisms: acutely declined levels of Gomafu in response to neuronal activation and Gomafu-mediated impaired alternative splicing through its binding directly to the two splicing factors QK1 and serine/arginine-rich splicing factor 1, which ultimately leads to the abnormal regulation of the two schizophrenia genes DISC-1 and ErbB4 (Barry et al., 2014; Liao et al., 2016), causing a decrease in the activity of parvalbumin interneurons (Chung et al., 2016). Soon after the identification of the association between lncRNA Gomafu and schizophrenia, the link of its genetic alterations with schizophrenia in different populations has been subsequently reported. Rao et al. (2015) have conducted an analysis on the genetic variants of lncRNA Gomafu in 1255 patients diagnosed with paranoid schizophrenia, compared to 1209 healthy individuals in a Chinese Han population, and found that the rs1894720 locus was significantly associated with paranoid schizophrenia (Rao et al., 2015). Aside from lncRNA Gomafu, there are other mental disease-related lncRNAs such as Evf2, BDNF-AS and DISC-2 that have been found to be associated with schizophrenia and have been reviewed previously (Merelo et al., 2015).

As the number of new schizophrenia-associated ncRNAs continues to grow and the variants within the ncRNA genes may directly correlate with schizophrenia, there is an urgent need for novel genetic tools that can be used to understand the functional role of these ncRNAs in schizophrenia and to correct the mutations as a novel approach for schizophrenia research and treatment. CRISPR/Cas9 is such a revolutionary technology that can be applied to work especially for this purpose.

## CRISPR/Cas9 for Editing Genomic Elements Targeting Non-Coding RNAs

The CRISPR/Cas9 system, originally well-documented as an adaptive immune defense mechanism in bacteria, is an emerging revolutionary yet feasible technique for precise genome editing of various organisms, including plants, animals, and even humans, so that specific changes in the genomic DNA stretches can be easily and precisely made (Bhaya et al., 2011; Jinek et al., 2012; Cho et al., 2013; Cong et al., 2013; Jiang et al., 2013; Mali et al., 2013; Doudna and Charpentier, 2014). The CRISPR/Cas9 system generally consists of two components: the Cas9 protein and the guide RNA (gRNA). Being guided by the gRNA, the Cas9 protein is recruited to the target site and is able to cut the genomic DNA at a specific site. The past years have witnessed the emergence of this innovative technology as well as dramatic progress in genomic DNA editing with the CRISPR/Cas9 system (Doudna and Charpentier, 2014). The technology has been applied to manipulate the genomic DNA elements targeting not only the coding regions but also the non-coding ones, from which small and long coding ncRNAs such as miRNAs and lncRNAs are encoded. It is particularly important to edit the genomic DNA elements targeting non-coding regions, since silencing these ncRNA genes with the current, conventional RNA interference (RNAi) technology has turned out to be challenging due to their resistance to the RNAi techniques (Zamore et al., 2000; Gilbert et al., 2013; Fatica and Bozzoni, 2014; Hilton et al., 2015). Recently, the obstacles have been overcome by the application of a modified CRISPR/Cas9 system, in which a lentiviral vector expressing two gRNAs are used simultaneously, with which the genomic DNA fragments ranging from 100 to 3000 bp in length have been manipulated and edited (Han et al., 2014; Aparicio-Prat et al., 2015). To date, a number of ncRNA genes in the genomic DNA have been successfully silenced, including miRNAs (miR21, miR29a) and lncRNAs (UCA1, MALAT1), with MALAT1 reduced by up to 98% inside human HCT116, Hela, and HEK293T cell lines after the promoter region of the MALAT1 gene was edited by the Double Excision CRISPR Knockout method (Aparicio-Prat et al., 2015; Ho et al., 2015; Chang et al., 2016). More detailed information about the miRNA and lncRNA genes successfully edited by the CRIPSR/Cas9 system in cell lines and entire animals were summarized in Tables 1, 2. This powerful genome-editing tool has brought a wealth of innovative applications and perspectives for both biological research and the treatment of diseases, including the complicated disease of schizophrenia. In the following sections, we offer a review of a number of successful examples of CRISPR/Cas9-mediated editing on the non-coding regions of genomic DNA in human cell lines and animal models as well as comment on the perspectives for both schizophrenia research and an eventual cure for schizophrenia. We also discuss the challenges of translating this advanced technology into a clinical therapy for schizophrenia and suggest strategies for removing the obstacles to accelerate the pace along the path from the bench to the bedside.

**Table 1:** **Summary of the miRNA genes successfully edited by the CRISPR/Cas9 system**.

miRNA	Chromosomal location	Cells/Animals tested	Results	Reference
miR-21	Chr17	Human HEK293, LNCaP, MCF-7 and HCT-116 cells	The miR-21 gene was knocked out with over 90% reduction of the miR-21 levels	Ho et al. (2015)
miR-29a	Chr7	Human HEK293 cells	Complete knock-out of the miR-29a gene	Ho et al. (2015)
miRNA cluster (miR-17a-1, miR-92a-1)	Chr1	Zebrafish	The deletion of this miRNA cluster gene from dre-miR-17a-1 to dre-miR-92a-2 was obtained in the zebrafish embryos injected with the CRISPR/Cas9 in combination with the gRNAs	Xiao et al. (2013)
miR-17	Chr13	Human HCT-116 and HT-29 cells	The miR-17 expression was decreased by up to 96% and the knock-down phenotype was stable in the human cells and mice	Chang et al. (2016)
		Mice		
miR-141	Chr12	Human HCT-116 and HT-29 cells	Nearly 96% reduction in the miR-141 levels was achieved with long-term stability of the knock-down phenotype and in control of crossing off-target effect on other members in the same family	Chang et al. (2016)
		Mice		

**Table 2:** **Summary of the lncRNA genes successfully edited by the CRISPR/Cas9 system**.

miRNA	Chromosomal location	Cells/Animals tested	Results	Reference
UCA1	Chr19	Human HCT-116 cells	After >5.6 kb in length for the UCA1 gene was edited, UCA1 RNA levels were reduced by about 90%	Ho et al. (2015)
MALAT1 or NEAT1	Chr11	Human cell lines: HEK293T, HCT-116, Hela and IMR90	Up to 98%reduction in MALAT1 RNA expression was achieved after the upstream and major promoters in the MALAT1 gene were deleted	Aparicio-Prat et al. (2015)
LncRNA-21A	Chr8	Human HCT-116 cells	475 bp for the lncRNA-21 A gene was targeted, wild-type lncRNA-21A was completely lost	Ho et al. (2015)
AK023948	Chr8	Human MCF-7 cells	The AK023948 gene was knocked out	Ho et al. (2015)
LncRNA Rian	Chr12	Mice	23 kb fragment in the lncRNA *Rian* gene was deleted, and a *Rian* knock-out mouse model with heritability of this large fragment deletion was established	Han et al. (2014)

*Abbreviations: UCA1, urothelial cancer associated 1; MALAT1, metastasis associated lung adenocarcinoma transcript 1; NEAT2, noncoding nuclear-enriched abundant transcript 2*.

## Perspective of Editing ncRNA Genes with CRISPR/Cas9 for Schizophrenia Research

The application of the novel CRISPR/Cas9 technology in schizophrenia-associated ncRNAs will usher in a new perspective for schizophrenia research to advance our understanding about the biological function for ncRNAs and to facilitate creating animal models with specific mutations or with the original form restored. Silencing or enhancing gene expression to lose or gain function has been well accepted in the study of a gene of interest. RNAi with specific small-interfering RNAs (siRNAs) is a commonly used approach to silence a desired gene encoded by the coding regions of genomic DNA, which usually occurs in the cytoplasm (Zamore et al., 2000). However, siRNAs designed to target ncRNA genes, including lncRNAs and miRNA genes, have been found to be inefficient mainly because many lncRNAs are located in the nucleus (Fatica and Bozzoni, 2014). In fact, it has been difficult to achieve successful knockdown of a desired lncRNA gene. Recently, scientists from independent research groups have applied a modified CRISPR/Cas9 system to target the ncRNA genes in the nucleus that has resulted in robust knockdown of a number of ncRNAs, including miRNAs in the zebrafish genome (Xiao et al., 2013), miRNAs in human cell lines, lncRNAs in a mouse model (Han et al., 2014) and lncRNAs in human cell lines (Ho et al., 2015), allowing the study of the biological roles of schizophrenia-associated ncRNAs in the pathogenesis of schizophrenia. The key feature of the modified CRISPR/Cas9 system is the use of dual gRNAs, which produce two cuts in specific sites and allow deletion of a larger fragment (Ho et al., 2015). With the modified CRISPR/Cas9 system, there is the possibility to manipulate any exon fragment of the ncRNAs and to explore the biological function of these ncRNA genes in schizophrenia. Since schizophrenia is a complex disorder that involves multiple genetic alterations of ncRNAs, the modified CRISPR/Cas9 approach will enable the disruption of these ncRNAs and will test if disruption of these ncRNA genes can cause schizophrenia. Apart from the silencing of ncRNA genes, the CRISPR/Cas9 system can also deliver regulatory components to the target genes and activate or upregulate target gene expression. Together with its silencing of the ncRNA gene, activation of the gene at the transcriptional level also empowers researchers to understand the biological role for a gene in the development and progression of schizophrenia. Apparent knockdown or activation of an ncRNA gene using the modified CRISPR/Cas9 system has couple of advantages: it is more effective than RNAi, and it is able to target multiple genes simultaneously. When it comes to the schizophrenia-associated ncRNAs miR137 and Gomafu, it is possible to target these two ncRNAs simultaneously and investigate if miRNA137 and Gomafu each alone or in combination affect the development and progression of schizophrenia.

The main bottleneck of schizophrenia research has been lack of translatable animal model system to prove causal relationship between genetic defects and pathophysiology of schizophrenia owing to not only difficulties in reproducing its prominent symptoms but also the tedious work required for creating animal models with specific genomic mutations. The CRISPR/Cas9 system brings a novel perspective for developing animal models for schizophrenia research due to a number of advantages. With the help of the CRISPR/Cas9 system, specific mutations of the target ncRNA gene can be introduced into the embryo and the normal form of the ncRNA gene can be restored in a rat or mouse embryo. The rat or mouse as well as its offspring will contain the mutation or restoration of the original form, which allows researchers to compare the symptoms directly, to determine how disruption of these genes affects the development and progression of schizophrenia, and to pinpoint the underlying molecular pathway in those animal models. The generated animal models can also be used to test the efficacy of medications or other potential therapeutic approaches in the treatment of schizophrenia. In fact, the animal model generated by traditional procedures usually takes up to 2 years to get the specific mutations in the offspring because multiple breeding steps are needed, while creating the animal model using the CRISPR/Cas9 approach only requires about 2 months and costs less. Furthermore, in combination with template DNAs and using multiple gRNAs, the CRISPR/Cas9 system is able to introduce a number of desired mutations into the embryo of an animal or their offspring, which is not likely to work out with other previous approaches.

## Perspective of Editing ncRNA Genes with CRISPR/Cas9 for the Treatment of Schizophrenia

Medications have been the cornerstone therapy for schizophrenia, and medications in combination with psychosocial interventions are widely used to manage schizophrenia. However, medications for schizophrenia usually cause serious adverse side effects; therefore, a large portion of patients with schizophrenia fail to take medications, leading to this devastating mental disease being uncontrolled. As a strong genetic component is involved in the pathogenesis of schizophrenia, multiple alterations in the genomic DNA of neurons have been implicated as causative factors, and fortunately great progress has been recently made in both identifying critical genomic regions and developing advanced genetic technologies, multiple alterations may be manipulated in the genomic DNA of neurons. As we discussed earlier, the genetic mutations in both miRNA and lncRNA genes have been directly linked to schizophrenia. The CRISPR/Cas9 system has provided a valuable tool to correct mutations not only in inheritable genetic diseases but also gene mutations in DNA genomes related to diseases, including ncRNAs genes. The CRISPR/Cas9 system, together with a donor DNA template, is required and delivered together with the CRISPR/Cas9 system into the recipients, and it has been successfully used to replace the mutations with substitutions in cells, plants, and entire animals to correct certain genomic DNA mutations. For example, a research group from Duke University has recently explored the application of the CRISPR/Cas9 system to treat Duchenne muscular dystrophy (DMD), a genetic disease with a debilitating mutation within one of the exons of the dystrophin gene (Nelson C. E. et al., 2016). For the first time, researchers have successfully treated a human disease in a living mouse model (Nelson C. E. et al., 2016) with the CRISPR/Cas9 gene editing technology. Meanwhile, similar results from two other research groups from Harvard University and the University of Texas are exciting as well (Long et al., 2016; Tabebordbar et al., 2016). These three independent research groups have demonstrated that correction of the dystropin gene, the consequential restoration of functional dystrophin, and the enhancement of muscle strength can be achieved after one of the exons in the dystrophin gene was reverted with the CRISPR/Cas9 technique by using different methods to deliver the CRISPR/Cas9 components (Long et al., 2016; Nelson C. E. et al., 2016; Tabebordbar et al., 2016).

In addition to DMD, a variety of other genetic diseases such as sickle-cell anemia and Alzheimer’s disease have been treated with the CRISPR/Cas9 technique by correcting the causative mutations and reverting the defect to the original form in the genomic DNA, and functional restoration has been gained (Huang et al., 2015; Sankaran and Weiss, 2015; Paquet et al., 2016). Recently, a research team from Sun-Yatsen University in Guangzhou, China, has explored, for the first time, the utilization of this novel CRISPR/Cas9 system to edit the thalassemia-causing gene in human embryos (Liang et al., 2015; Callaway, 2016). As a result, a number of human embryos have successfully gained the corrected form of the target gene. More recently, the successful application of the CRISPR/Cas9 gene editing technique to human induced pluripotent stem cells (IPSCs) have been reported to generate disease model (Horii et al., 2013) and to treat effectively a number of diseases, including epi-dermolysis bullosa (Osborn et al., 2013), b-thalassemia (Ma et al., 2013), a1-antitrypsin deficiency (Choi et al., 2013), AIDS (Ye et al., 2014), and Niemann-Pick Type C (Maetzel et al., 2014), DMD (Li et al., 2014). Therefore, this novel approach has provided enormous promise for gene therapy of many other forms of diseases including schizophrenia.

Despite the use of genome editing technologies including zinc finger nucleases, TALENS, and CRISPR/Cas9 has opened up the possibility of *in vivo* genome editing therapy. Unlike peripheral nerve disorder such as DMD, brain and mental disorders (i.e., Parkinson’s disease and schizophrenia) have a unique challenge in clinical settings. AAVs vectors, which have been recently approved by FDA for a clinical use, have effectively delivered the nucleases and other components to a variety of tissues including brain. Compared to the other genome editing approaches, the gRNA sequence used in the CRISPR/Cas9 system is more easily to be altered than for TALEs, and a much shorter gRNA and multiple gRNAs can be used to result in multiple DNA cuts (Hsu et al., 2014; Cox et al., 2015) suggesting more promising for the use of CRISPR/Cas9 as a potential therapeutic intent. Most recently, Deverman et al. (2016) reported that they developed a harmless virus, namely AAV-PHP.B, to across the blood brain barrier to successfully deliver treatment to the brain.

These exciting results in living mammals and human cells show great potential for treating human diseases by translating this CRISPR/Cas9 system to a novel therapeutic approach to treat genetically related diseases in humans, including schizophrenia and perhaps other genetically related mental disorders due to the overlapping genetic alterations of schizophrenia with other mental illnesses.

## Challenges and Future Directions

Despite the remarkable progress, the CRISPR/Cas9 system is still immature and has some limitations and challenges to overcome. Technical improvement is needed, while ethical issues require resolution as well. There are a couple of technical challenges, including specificity, efficiency of the system and penetration of the blood-brain barrier for delivery of the components in the CRISPR/Cas9 system. When a potential therapeutic approach to treat brain and mental disorders is discovered, the blood-brain barrier always poses an obstacle for the drugs to reach the brain cells. The components themselves in the CRISPR/Cas9 system cannot pass through the blood-brain barrier. In an effort to deliver the CRISPR/Cas9 components, a few inactive genetically engineered viruses have been successfully used as a carrier that can be packed with the CRISPR/Cas9 components, cross the blood-brain barrier, and finally unload those molecules in the brain cells. In spite of having no capacity to cause disease, further improvement of the efficiency and the evaluation of long-term safety are needed. In addition to the above-mentioned technical concerns, there are ethical issues as well for manipulating genomic DNA in human sperm, eggs and embryos, mainly because of limited knowledge regarding the CRISPR/Cas9 system and the long-term impact of genetic disruption and off-targeting on future generations (Krishan et al., 2016). To date, ncRNAs have not been reported to express proteins; Nelson B. R. et al. (2016) have recently reported that a lnRNA encodes a peptide with the ability to enhance SERCA activity in muscle. With the possibility of unidentified proteins being translated by ncRNAs, it is uncertain whether the proteins may exert any unfavorable, even disease-related activity. All of these new questions require answers; therefore, along with other issues, there is still a long way to go before this revolutionary technology is translated into a clinical cure for schizophrenia and possibly other mental diseases as well due to significant overlaps of the risk genes shared by different forms of psychiatric disorders.

## Author Contributions

CZ: conceptional design and writing of the draft manuscript. WH: conceptional design and writing of the final manuscript. LH, CL, CC and XL collected and examined the enrolled articles in this review.

## Funding

This review was funded by Jiangsu Haosen pharmaceutical Limited by Share Ltd (2016-Young scholar support project to CZ).

## Conflict of Interest Statement

The authors declare that the research was conducted in the absence of any commercial or financial relationships that could be construed as a potential conflict of interest. The reviewer DT and handling Editor declared their shared affiliation, and the handling Editor states that the process nevertheless met the standards of a fair and objective review.
